# Toll-Like Receptor 2 Impairs Host Defense in Gram-Negative Sepsis Caused by *Burkholderia* *pseudomallei* (Melioidosis)

**DOI:** 10.1371/journal.pmed.0040248

**Published:** 2007-07-31

**Authors:** W. Joost Wiersinga, Catharina W Wieland, Mark C Dessing, Narisara Chantratita, Allen C Cheng, Direk Limmathurotsakul, Wirongrong Chierakul, Masja Leendertse, Sandrine Florquin, Alex F de Vos, Nicholas White, Arjen M Dondorp, Nicholas P Day, Sharon J Peacock, Tom van der Poll

**Affiliations:** 1 Center for Infection and Immunity Amsterdam, Academic Medical Center, Amsterdam, The Netherlands; 2 Center for Experimental and Molecular Medicine, Academic Medical Center, Amsterdam, The Netherlands; 3 Wellcome Trust, Mahidol University, Bangkok, Thailand; 4 Menzies School of Health Research, Darwin, Australia; 5 Department of Pathology, Academic Medical Center, Amsterdam, The Netherlands; 6 Center for Clinical Vaccinology and Tropical Medicine, University of Oxford, Oxford, England; Brown University School of Medicine, United States of America

## Abstract

**Background:**

Toll-like receptors (TLRs) are essential in host defense against pathogens by virtue of their capacity to detect microbes and initiate the immune response. TLR2 is seen as the most important receptor for gram-positive bacteria, while TLR4 is regarded as the gram-negative TLR. Melioidosis is a severe infection caused by the gram-negative bacterium, *Burkholderia pseudomallei,* that is endemic in Southeast Asia. We aimed to characterize the expression and function of TLRs in septic melioidosis.

**Methods and Findings:**

Patient studies: 34 patients with melioidosis demonstrated increased expression of CD14, TLR1, TLR2, and TLR4 on the cell surfaces of monocytes and granulocytes, and increased *CD14, TLR1, TLR2, TLR4, LY96* (also known as *MD-2*), *TLR5,* and *TLR10* mRNA levels in purified monocytes and granulocytes when compared with healthy controls. In vitro experiments: Whole-blood and alveolar macrophages obtained from TLR2 and TLR4 knockout (KO) mice were less responsive to B. pseudomallei in vitro, whereas in the reverse experiment, transfection of HEK293 cells with either TLR2 or TLR4 rendered these cells responsive to this bacterium. In addition, the lipopolysaccharide (LPS) of B. pseudomallei signals through TLR2 and not through TLR4. Mouse studies: Surprisingly, TLR4 KO mice were indistinguishable from wild-type mice with respect to bacterial outgrowth and survival in experimentally induced melioidosis. In contrast, TLR2 KO mice displayed a markedly improved host defenses as reflected by a strong survival advantage together with decreased bacterial loads, reduced lung inflammation, and less distant-organ injury.

**Conclusions:**

Patients with melioidosis displayed an up-regulation of multiple TLRs in peripheral blood monocytes and granulocytes. Although both TLR2 and TLR4 contribute to cellular responsiveness to B. pseudomallei in vitro, TLR2 detects the LPS of *B. pseudomallei,* and only TLR2 impacts on the immune response of the intact host in vivo. Inhibition of TLR2 may be a novel treatment strategy in melioidosis.

## Abstract 2

Willem Wiersinga and colleagues find up-regulation of multiple Toll-like receptors (TLRs) in peripheral blood cells of patients with melioidosis. However, only TLR2 had an effect on the immune response in a mouse model.

## Editors' Summary

**Background.:**

Melioidosis is a severe tropical infection caused by the bacterium Burkholderia pseudomallei. This soil-dwelling pathogen (disease-causing organism) enters the body through cuts, by swallowed contaminated water, or by inhaled contaminated dust. Here, it can cause a severe lung infection or spread into the blood stream and around the body, where it causes widespread inflammation (sepsis) and organ failure. Untreated septic melioidosis is usually fatal. Even with antibiotic therapy, half the people who develop it in Thailand (a hot spot for melioidosis) die. B. pseudomallei is a “gram-negative” bacterium. That is, it is surrounded by a membrane that stops it taking up a stain used to detect bacteria. This membrane contains a molecule called lipopolysaccharide (LPS). Proteins on immune system cells called Toll-like receptors (TLRs), of which there are many, recognize LPS and other surface molecules common to different pathogens and tell the cells to make cytokines. These cytokines stimulate the immune system to kill the pathogen but also cause inflammation, the underlying problem in septic melioidosis and other forms of sepsis. In other words, TLRs are two-edged swords—they provide an essential first-line defense against pathogens, but cause life-threatening inflammation if overstimulated.

**Why Was This Study Done?:**

It isn't known which TLRs are involved in melioidosis. TLR4 normally detects LPS, but the surface of B. pseudomallei also carries molecules that interact with TLR2. Understanding how B. pseudomallei interacts with TLRs might suggest new, more effective ways to treat septic melioidosis. Better remedies for this disease are badly needed because, as well as the infections it causes in the community, the US Centers for Disease Control and Prevention has identified B. pseudomallei as a potential bioterrorism agent. In this study, the researchers have characterized the expression and function of TLRs in septic melioidosis using human, in vitro (test tube), and animal approaches.

**What Did the Researchers Do and Find?:**

The researchers isolated monocytes and granulocytes (immune system cells involved in first-line defenses against pathogens) from patients with melioidosis and from healthy people. The patients' cells made more TLR1, TLR2, TLR4, and CD14 (a protein that enhances the activation of immune system cells by LPS) than those of the healthy controls and more of the mRNAs encoding several other TLRs. Next, the researchers tested the ability of heat-killed B. pseudomallei to induce the release of TNFα (a cytokine produced in response to TLR signaling) from macrophages (immune system cells that swallow up pathogens) isolated from wild-type mice and from mice lacking TLR2 or TLR4. Macrophages isolated from wild-type mice made more TNFα than those from TLR2- or TLR4-deficient mice. In addition, a human kidney cell line engineered to express CD14/TLR2 or CD14/TLR4 but not the parent cell line released IL8 (another cytokine) when stimulated with heat-killed B. pseudomallei. Other experiments in these human cell lines showed that LPS purified from B. pseudomallei signals through TLR2 but not through TLR4. Finally, the researchers tested the ability of TLR2- and TLR4-deficient mice to survive after infection with live B. pseudomallei. Compared with TLR4-deficient or wild-type mice, the TLR2-deficient mice had a strong survival advantage, a lower bacterial load, reduced lung inflammation, and less organ damage.

**What Do These Findings Mean?:**

These findings show that people with melioidosis have increased expression of several TLRs, any one of which might cause the sepsis associated with *B. pseudomallei* infection. The in vitro findings indicate that TLR2 and TLR4 both contribute to the responsiveness of immune cells to B. pseudomallei in test tubes, but that only TLR2 detects the LPS of this bacterium. This unexpected result—TLR4 normally responds to LPS—might indicate that there is something unique about the LPS of B. pseudomallei. Finally, the survival of TLR2-deficient mice after infection with B. pseudomallei suggests that TLR2-mediated dysregulation of the immune system in response to invasive B. pseudomallei might cause septic melioidosis. Although these results need confirming in people, they suggest that inhibition of TLR2 in combination with antibiotic therapy might improve outcomes for people with melioidosis.

**Additional Information.:**

Please access these Web sites via the online version of this summary at http://dx.doi.org/10.1371/journal.pmed.0040248.

- Information is available from the US Centers for Disease Control and Prevention on melioidosis (in English and Spanish)
- The UK Health Protection Agency provides information for the public and health professionals on melioidosis
- Wikipedia has pages on melioidosis and on Toll-like receptors (note: Wikipedia is a free online encyclopedia that anyone can edit; available in several languages)
- The MedlinePlus encyclopedia contains a page on sepsis (in English and Spanish)

## Introduction

The recently discovered family of Toll-like receptors (TLRs), which are conserved across many species, has emerged as an important first line of defense against invading pathogens. They detect host invasion by pathogens, initiate immune responses, and form the crucial link between the innate and adaptive immune systems [1,2]. Upon the first encounter with a pathogen, TLRs recognize conserved motifs termed pathogen-associated molecular patterns (PAMPs) [1,2]. In general, the immune activation that follows the interaction between TLRs and PAMPs is sufficient to eliminate the wide variety of pathogens that daily invade the human body. However, in the case of sepsis, these TLR-mediated inflammatory responses may exceed the threshold below which immune system homeostasis is maintained and thereby cause harm.

In Southeast Asia and northern Australia the gram-negative soil-dwelling bacillus Burkholderia pseudomallei is an important cause of community-acquired sepsis and sepsis-related mortality [3,4]. More than half of the cases of melioidosis, as this severe infection is named, commonly presents with pneumonia with bacterial dissemination to distant sites [5]. The mortality of primary disease is 50% in northeast Thailand and around 20% in the higher-technology setting of northern Australia [6,7]. Reported cases are likely to represent “the tip of the iceberg,” since confirmation of disease depends on bacterial isolation, a technique that is not available in large areas of the world [3,8]. Interest in disease pathogenesis of B. pseudomallei has increased following its classification as a category B disease/agent of bioterrorism by the US Centers for Disease Control and Prevention (http://www.bt.cdc.gov/agent/agentlist.asp).

It is likely that TLRs contribute to host defense against *B. pseudomallei.* In general, TLR4 is considered of utmost importance for host defense against gram-negative infection by virtue of its capacity to sense lipopolysaccharide (LPS) present in the outer membrane of gram-negative bacteria and to relay LPS effects into the cellular interior [9]. Besides TLR4, TLR2 may play a role in the host response to *B. pseudomallei,* given that this bacterium expresses several PAMPs that can interact with TLR2, including lipopeptides and peptidoglycan. However, these assumptions remain speculative since the expression and function of these TLRs have not been studied in melioidosis thus far.

In the present study we aimed to characterize the expression and function of TLRs in sepsis caused by B. pseudomallei and found that, although both TLR2 and TLR4 contribute to cellular responsiveness to B. pseudomallei in vitro, only TLR2 impacts on the immune response of the intact host in vivo.

## Methods

### Human Studies

#### Patients.

Blood samples were taken from 34 patients with sepsis caused by B. pseudomallei. Patients were prospectively recruited over a 4 mo period after admission to Sapprasithiprasong Hospital, a 1,000-bed government hospital located in Ubon Ratchathani, northeast Thailand. Sepsis caused by melioidosis was defined by the presence of (1) culture-proven infection with B. pseudomallei and (2) systemic inflammatory response syndrome (SIRS) as indicated by three or more of the following criteria: a core temperature of ≥ 38 °C or ≤ 36 °C; a heart rate of ≥ 90 beats/min; a respiratory rate of ≥ 20 breaths/min or a Paco_2_ of ≥ 32 mm Hg or the use of mechanical ventilation for an acute respiratory process; a white-cell count of ≥ 12 × 10^9^/l or ≤ 4 × 10^9^/l or a differential count showing > 10% immature neutrophils [10]. Exclusion criteria were the use of dialysis and/or immunosuppressive therapy, known disorders of coagulation, and concomitant infection with HIV. Blood samples were drawn within 36 h after start of antimicrobial therapy. When possible, an additional blood sample was drawn from patients who recovered after 2 wk of treatment. 32 healthy individuals, who were recruited from the Sapprasithiprasong Hospital blood bank as blood donors, served as a control population. The study was approved by both the Ministry of Public Health, Royal Government of Thailand and the Oxford Tropical Research Ethics Committee, University of Oxford, England and written informed consent was obtained from all study participants or their attending relatives.

#### RNA analysis using multiplex ligation-dependent probe amplification.

Heparinized blood samples were drawn from the antecubital vein and immediately put on ice. Leukocytes were isolated using erythrocyte lysis buffer. Monocyte- and granulocyte-enriched populations were isolated using Polymorphprep (Axis-Shield, http://www.axis-shield.com/). Monocyte and granulocyte fractions were > 98% pure as determined by their scatter pattern on flow cytometry. After isolation leukocytes, monocytes, and granulocytes were dissolved in Trizol (Invitrogen, http://www.invitrogen.com/) and stored at −80 °C until used for RNA isolation. RNA was isolated and analyzed by multiplex ligation-dependent probe amplification (MLPA) as described [11–14] using a TLR-specific kit developed in collaboration with MRC-Holland (http://www.mlpa.com/) for the simultaneous detection of 17 mRNA molecules; four different probe sets were generated for TLR4 corresponding with four different TLR4 splice variants (R01 to R04) identified in the National Center for Biotechnology Information (NCBI) (http://www.ncbi.nlm.nih.gov); similarly, two different probe sets for TLR8 were generated corresponding with two different TLR8 splice variants (R01 and R02). We recently published the specific oligonucleotides used for all TLR probe sets [15]. All samples were tested with the same batch of reagents. The levels of mRNA for each gene were expressed as a normalized ratio of the peak area divided by the peak area of the *beta-2 microglobulin (B2M)* gene, resulting in the relative abundance of mRNAs of the genes of interest [11–14].

#### FACS analysis.

CD14 and TLR cell surface expression on peripheral monocytes and granulocytes was determined by flow cytometry using fluorochrome-conjugated mouse anti-human CD14 (BD Biosciences, http://www.bdbiosciences.com/), TLR1, TLR2, and TLR4 (all eBioscience, http://www.ebioscience.com/) antibodies in accordance with the manufacturer's recommendations. Granulocytes were defined according to their scatter pattern and monocytes according to their scatter pattern and CD14 positivity. To correct for nonspecific staining, appropriate isotype control antibodies (BD Biosciences) were used. Samples were analyzed directly after sample collection by flow cytometry using FACScan (BD Biosciences).

### Mouse and In Vitro Studies

#### Mice.

Pathogen-free 8- to 10-wk-old wild-type (WT) C57BL/6 mice were purchased from Harlan Sprague Dawley (http://www.harlan.com/). TLR2 knockout (KO) mice [16] and TLR4 KO mice [17] backcrossed six times to a C57BL/6 background were generously provided by Dr. Shizuo Akira, Osaka University, Japan. Age- and sex-matched animals were used in all experiments. The Animal Care and Use of Committee of the University of Amsterdam approved all experiments.

#### Preparation of alveolar macrophages.

Alveolar macrophages were harvested from TLR2 KO, TLR4 KO, and WT mice by bronchoalveolar lavage (*n* = 5–8 per strain) as described [18,19].The trachea was exposed through a midline incision and cannulated with a sterile 22-gauge Abbocath-T catheter (Abbott, http://www.abbott.ie/). Bronchoalveolar lavage was performed by instilling three 0.5 ml aliquots of sterile saline. Total cell numbers were counted from each sample using a hemacytometer. Cells were resuspended in RPMI 1640 containing 1 mM pyruvate, 2 mM L-glutamine, penicillin/ streptomycin (100 units penicillin, 100 g streptomycin), and 10% FCS in a final concentration of 1 × 10^4^ cells/100 μl. Cells were then cultured in 96-well microtiter plates (Greiner, http://www.greinerbioone.com/) for 2 h and washed with RPMI 1640 to remove nonadherent cells. Adherent monolayer cells were stimulated with washed (three times in normal saline), heat-killed B. pseudomallei (clinical isolate strain 1026b [20,21]; 1 × 10^5^ colony-forming units (CFU)/ml) or RPMI 1640 for 16 h. Supernatants were collected and stored at −20 °C until assayed for tumor necrosis factor α (TNFα).

#### LPS extraction from B. pseudomallei 1026.

LPS was extracted from B. pseudomallei 1026b [22]. Bacteria from frozen stock were inoculated into 5 ml of LB broth and incubated overnight at 37 °C in air with shaking at 200 rpm. This starting culture was streaked onto 50 LB agar plates per strain. Plates were incubated at 37 °C in air for 2 d, after which bacterial colonies were scraped from plates and resuspended in 100 ml of sterile distilled water. LPS extraction using a hot aqueous-phenol method was performed as described by Brett et al. [23]. Purified B. pseudomallei LPS was characterized by SDS-PAGE and silver staining [24].

#### HEK cells.

Human embryonic kidney (HEK) 293 cells stably expressing CD14, CD14−TLR4, or CD14−TLR2, kindly provided by Dr. Douglas Golenbock, University of Massachusetts Medical School, Worcester, Massachusetts, have been described [25,26]. Cells were stimulated with LPS from B. pseudomallei 1026b (100 ng/ml), heat-killed B. pseudomallei (5 × 10^7^ CFU/ml), or RPMI for 6 h. Supernatants were harvested and stored at −20 °C until assayed for interleukin (IL) 8.

#### Experimental infection.

For preparation of the inoculum, B. pseudomallei strain 1026b was used (this strain was isolated in Sappasithiprasong Hospital in 1993 from a blood culture from a septic 29-y-old female rice farmer, who presented with bacteremia with soft tissue, skin, joint, and spleen involvement [20,21]). Stock bacteria were streaked from frozen aliquots into 50 ml Luria broth (Difco) for overnight incubation at 37 °C in a 5% CO_2_ incubator. Thereafter, a 1 ml portion was transferred to fresh Luria broth and grown for approximately 5 h to midlogarithmic phase. Bacteria were harvested by centrifugation at 1500*g* for 15 min, washed, and resuspended in sterile isotonic saline at a concentration of 5 × 10^2^ CFU/50 μl, as determined by plating serial 10-fold dilutions on blood agar plates. Pneumonia was induced by intranasally inoculating mice with 50 μl (5 × 10^2^ CFU) of bacterial suspension. For this procedure, mice were lightly anesthetized by inhalation of isofluorane (Upjohn).

#### Determination of bacterial outgrowth.

At 24, 48, and 72 h after infection, mice were anesthetized with Hypnorm (Janssen Pharmaceutica, http://www.janssenpharmaceutica.be/; active ingredients, fentanyl citrate and fluanisone) and midazolam (Roche, http://www.roche.com/) and sacrificed by bleeding from the inferior vena cava. The lungs and spleens were harvested and homogenized at 4 °C in four volumes of sterile saline using a tissue homogenizer (Biospec Products, http://www.biospec.com/). CFUs were determined from serial dilutions of organ homogenates and blood, plated on blood agar plates and incubated at 37 °C at 5% CO_2_ for 16 h before colonies were counted.

#### Preparation of lung tissue for cytokine measurements.

For cytokine measurements, lung homogenates were diluted 1:2 in lysis buffer containing 300 mM NaCl, 30 mM Tris (pH 7.4), 2 mM MgCl_2_, 2 mM CaCl_2,_ 1% Triton X-100, and pepstatin A, leupeptin, and aprotinin (all 20 ng/ml) and incubated at 4 °C for 30 min. Homogenates were centrifuged at 1,500*g* at 4 °C for 15 min, and supernatants were stored at −20 °C until assays were performed.

#### Assays.

Human IL8 was measured by ELISA (Biosource, http://www.biosource.com/). Mouse TNFα, IL6, and IL10 were measured by cytometric bead array multiplex assay (BD Biosciences) in accordance with the manufacturer's recommendations. Aspartate aminotransferase (ASAT) and alanine aminotransferase (ALAT) were determined with commercially available kits (Sigma-Aldrich, http://www.sigmaaldrich.com), using a Hitachi analyzer (Roche) according to the manufacturer's instructions. Immunostaining for TLR4 on blood cells and whole lung-cell suspensions was performed using directly labeled antibodies against GR-1 (GR-1 FITC; Pharmingen) and TLR4 (TLR4-AlexaFluor, all Bioscience) and a biotin-labeled antibody against F4/80 (Serotec, http://www.ab-direct.com/) in combination with streptavidin allophycocyanine. All antibodies were used in concentrations recommended by the manufacturer. After staining, cells were fixed in 2% paraformaldehyde. TLR4 mean fluorescence intensity (MFI) was measured in the Gr-1-high gate (granulocytes), sidescatter low and F4/80 positive (monocytes), and sidescatter high and F4/80 positive (macrophages) gated populations.

#### Pathology.

Lungs and spleens for histology were harvested after infection, fixed in 10% formalin and embedded in paraffin. Sections of 4 μm were stained with hematoxylin–eosin, and analyzed by a pathologist who was blinded as to groups. To score lung inflammation and damage, the entire lung surface was analyzed with respect to the following parameters: surface with pneumonia, necrosis and/or formation of abscess, interstitial inflammation, endothelialitis, bronchitis, edema, thrombus formation, and pleuritis. Each parameter was graded on a scale of 0 to 4: 0, absent; 1, mild; 2, moderate; 3, severe; and 4, very severe. The total “lung inflammation score” was expressed as the sum of the scores for each parameter, the maximum being 32. Spleen sections were scored on inflammation, necrosis/abscess formation, and thrombus formation using the scale given above. The maximum total spleen inflammation score was 12. Granulocyte staining was done exactly as described previously [27,28].

### Statistical Analysis

Values are expressed as mean ± standard error of the mean (SEM). Differences between groups were analyzed by Mann-Whitney U test or Kruskal-Wallis analysis with Dunn post hoc test where appropriate. For survival analysis, Kaplan-Meier analysis followed by log-rank test was performed. These analyses were performed using GraphPad Prism version 4.00, GraphPad Software (http://www.graphpad.com/). Values of *p* < 0.05 were considered statistically significant.

## Results

### Patient Characteristics

A total of 34 patients with sepsis caused by B. pseudomallei (mean age 52 y, range 18–86 y; 50% male) and 32 healthy control participants (mean age 41 y, range 21–59 y; 71% male) were enrolled. B. pseudomallei was cultured from body material from all patients: blood cultures were positive for *B. pseudomallei* in 21 patients (61.7%), throat swab or tracheal suction in 13 patients (38.0%), sputum in seven patients (21.0%), pus from abscesses in seven patients (21.0%), and urine in five patients (14.7%). The overall patient mortality was 44%.

### Increased *CD14, LY96 (MD-2), TLR1, TLR2, TLR3, TLR4, TLR5, TLR8,* and *TLR10* mRNA Levels in Peripheral Blood Cells of Patients with Septic Melioidosis

To obtain a first insight into TLR gene expression during melioidosis, we quantified TLR mRNAs in blood leukocytes harvested from patients with septic melioidosis and healthy controls. MLPA was performed on RNA isolated from whole blood leukocytes as well as from monocyte- and granulocyte-enriched (> 98% pure) populations. Patients with melioidosis displayed elevated mRNA levels for most TLRs and TLR-related proteins in unfractionated leukocytes, monocytes, and granulocytes (Table 1; data of unfractionated leukocytes not shown). Specifically, melioidosis was associated with enhanced expression of monocyte and granulocyte mRNAs encoding TLR1, TLR2, TLR4 (R01 and R04), TLR5, TLR8 (R01 and R02)*,* and the TLR4 co-receptors LY96 (previously known as MD-2) and CD14. Of note, *TLR10* mRNA was up-regulated only in unfractionated leukocytes (unpublished data) and granulocytes, but not in monocytes (Table 1). The mRNAs encoding TLR3, TLR7 (both similar in patients and controls), and TLR9 (undetectable in patients and controls) were not influenced by melioidosis.

**Table 1:**
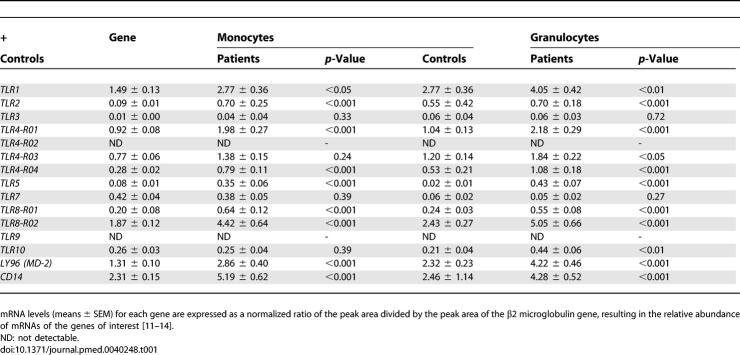
Toll-Like Receptor mRNA Expression in Peripheral Blood Monocytes and Granulocytes of Patients with Septic Melioidosis (*n* = 34) and Healthy Controls (*n* = 32)

### Increased Expression of CD14, TLR1, TLR2, and TLR4 on the Cell Surfaces of Circulating Monocytes and Granulocytes of Patients with Septic Melioidosis

**Figure 1:**
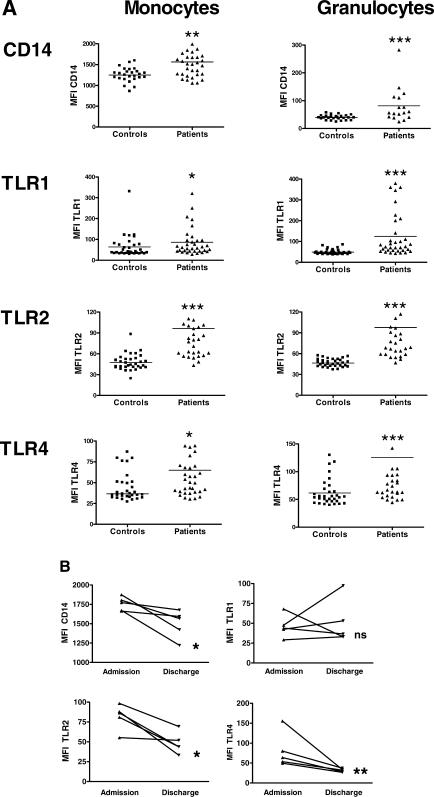
Increased Expression of CD14, TLR1, TLR2, and TLR4 on Peripheral Blood Monocytes and Granulocytes of Patients with Melioidosis (A) Increased expression of CD14, TLR1, TLR2, and TLR4 on peripheral blood monocytes and granulocytes of patients with severe melioidosis (triangles; *n* = 34) and healthy controls (squares; *n* = 32). Horizontal lines indicate median MFI. (B). Patients (*n* = 5) who survived after 2 wk of intensive treatment showed normalization of CD14, TLR2, and TLR4, but not of TLR1 expression on the cell surface of monocytes at discharge. ns, not significant. *p < 0.05; **p < 0.01; ***p < 0.001.

To determine the effect of B. pseudomallei sepsis on TLR protein expression at the surface of peripheral blood cells, we compared the expression of CD14, TLR1, TLR2, and TLR4 on circulating monocytes and granulocytes of patients with melioidosis and healthy controls using FACS analysis. Relative to controls, patients displayed higher cell-associated levels of CD14, TLR1, TLR2, and TLR4 on both monocytes and granulocytes (Figure 1A). In addition, in five patients from whom blood could be obtained after recovery (14 d after admission), monocyte expression of CD14, TLR2, and TLR4 decreased during follow-up, whereas TLR1 levels did not change in a consistent way (Figure 1B).

### TLR2 and TLR4 Contribute to Cellular Responsiveness to B. pseudomallei In Vitro

**Figure 2:**
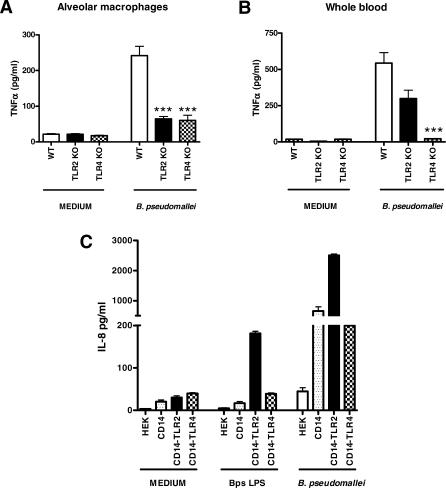
TLR2 and TLR4 Contribute to Cellular Responsiveness to B. pseudomallei In Vitro (A and B) Freshly isolated alveolar macrophages (A) and whole blood (B) of WT, TLR2 KO, and TLR4 KO mice (*n* = 8 per group) were incubated with RPMI 1640 (control) or heat-killed B. pseudomallei (equivalent 10^5^ CFU; target:effector ratio 1:10) for 16 h before TNFα production was measured. (C) HEK293 cells stably transfected with CD14, CD14/TLR2, or CD14/TLR4 were incubated for 6 h with medium, LPS of B. pseudomallei 1026, or heat-killed B. pseudomallei before measurement of IL8 in the supernatant. Data are mean ± SEM (*n* = 4). ***p < 0.001.

Having established that in particular TLR2 and TLR4 become up-regulated at the surface of blood leukocytes in patients with severe melioidosis, we sought to obtain insights into the function of these TLRs in melioidosis by testing the requirement of TLR2 and TLR4 signaling upon first encounter between the bacterium and the host. We first tested the capacity of alveolar macrophages and whole blood harvested from WT, TLR2 KO, or TLR4 KO mice to release TNFα upon stimulation with heat-killed B. pseudomallei (effector:target ratio 1:10). Whole blood and alveolar macrophages obtained from TLR2 KO or TLR4 KO mice released less TNFα than did alveolar macrophages and blood from WT mice upon stimulation with B. pseudomallei in vitro (Figure 2A and 2B). Consistent with these KO data, HEK293 cells stably transfected with either CD14/TLR2 or CD14/TLR4 responded to B. pseudomallei as reflected by a 10× to 100× increase in the release of IL8 into the supernatant (Figure 2C). These data suggest that both TLR2 and TLR4 contribute to cellular responsiveness to B. pseudomallei in vitro.

### The LPS of B. pseudomallei Signals through TLR2

LPS has been implicated as playing a major role in the induction of an innate immune response to gram-negative bacteria. Although TLR4 is considered the LPS receptor, LPS derived from some bacteria are recognized by TLR2. We therefore were interested to determine whether LPS from B. pseudomallei is recognized by TLR2 or TLR4. Remarkably, using HEK293 cells stably transfected with either CD14/TLR2 or CD14/TLR4, we found that purified LPS of B. pseudomallei 1026b signals through TLR2 and not through TLR4 (Figure 2C).

### TLR2 KO Mice, but Not TLR4 KO Mice, Are Protected from B. pseudomallei-Induced Death

**Figure 3:**
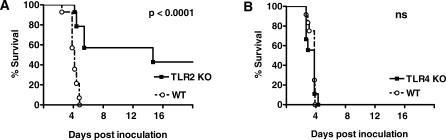
Survival of TLR2 KO Mice, but Not of TLR4 KO Mice, Is Enhanced during Experimental Melioidosis (A) Survival after intranasal inoculation with B. pseudomallei in WT (open circles), TLR2 KO mice (closed squares) and (B) TLR4 KO mice (closed squares). Mortality was assessed twice daily for 6 wk. *n* = 12 per group. *p*-Value indicates the difference between TLR2 KO and WT mice. ns, not significant.

Having established that TLR2 and TLR4 take part in the recognition of B. pseudomallei by immune cells in vitro, we next investigated the involvement of these receptors in the outcome of melioidosis. TLR2 KO, TLR4 KO, and WT mice were intranasally infected with B. pseudomallei and followed for 6 wk. All WT and TLR4 KO mice died within 5 d after inoculation. However, mortality was delayed and reduced among TLR2 KO mice, of which 40% survived until the end of the 6 wk observation period (*p* < 0.0001 for the differences between both mouse strains; Figure 3).

### TLR2 KO Mice, but Not TLR4 KO Mice, Show Reduced Growth of B. pseudomallei In Vivo

**Figure 4:**
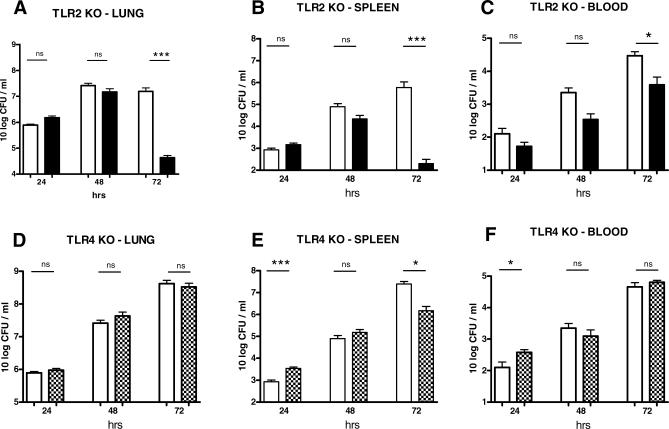
Bacterial Loads in Lungs, Spleen, and Blood TLR2 KO mice demonstrate strongly reduced bacterial loads at 72 h after infection in lungs (A), spleen (B), and blood (C). TLR4 KO mice showed a modestly enhanced outgrowth at 24 h after infection in spleen (E) and blood (F) but not in lungs (D). Data are mean ± SEM (*n* = 8 per group) at 24, 48, and 72 h after inoculation with B. pseudomallei in WT mice (open bars), TLR2 KO mice (black bars), and TLR4 KO mice (crosshatched bars). *p < 0.05; ***p < 0.0001. ns, not significant.

To obtain insight into the mechanisms underlying the reduced mortality of TLR2 KO mice during experimental melioidosis, we infected WT, TLR2 KO, and TLR4 KO mice with B. pseudomallei and sacrificed the mice after 24, 48, and 72 h (i.e., directly before the first predicted death in WT mice) to determine bacterial loads in lungs (the primary site of the infection), blood, and spleen (to evaluate extent of bacterial dissemination) (Figure 4). Relative to WT mice, TLR2 KO mice displayed strongly reduced bacterial loads in lungs, blood, and spleen at 72 h; this was also observed at the 48 h time point, although the difference did not reach statistical significance. In TLR2 KO mice that survived 6 wk after inoculation almost all bacteria were effectively cleared (unpublished data). TLR4 KO mice demonstrated higher bacterial loads only in spleen (*p* < 0.001) and blood (*p* < 0.05) at 24 h after infection; however, at later time points bacterial burdens did not differ significantly in TLR4 KO and WT mice in all body compartments except for the spleen at 72 h, revealing modestly fewer B. pseudomallei CFU in TLR4 KO mice (*p* < 0.001). Together, these data suggest that TLR4 may play a minor role in limiting the early dissemination of B. pseudomallei from the lungs and, more strikingly, that the presence of TLR2 facilitates the growth of this pathogen in lungs and the subsequent spread to distant body sites.

### TLR2 KO Mice Demonstrate Reduced Lung Inflammation

**Figure 5:**
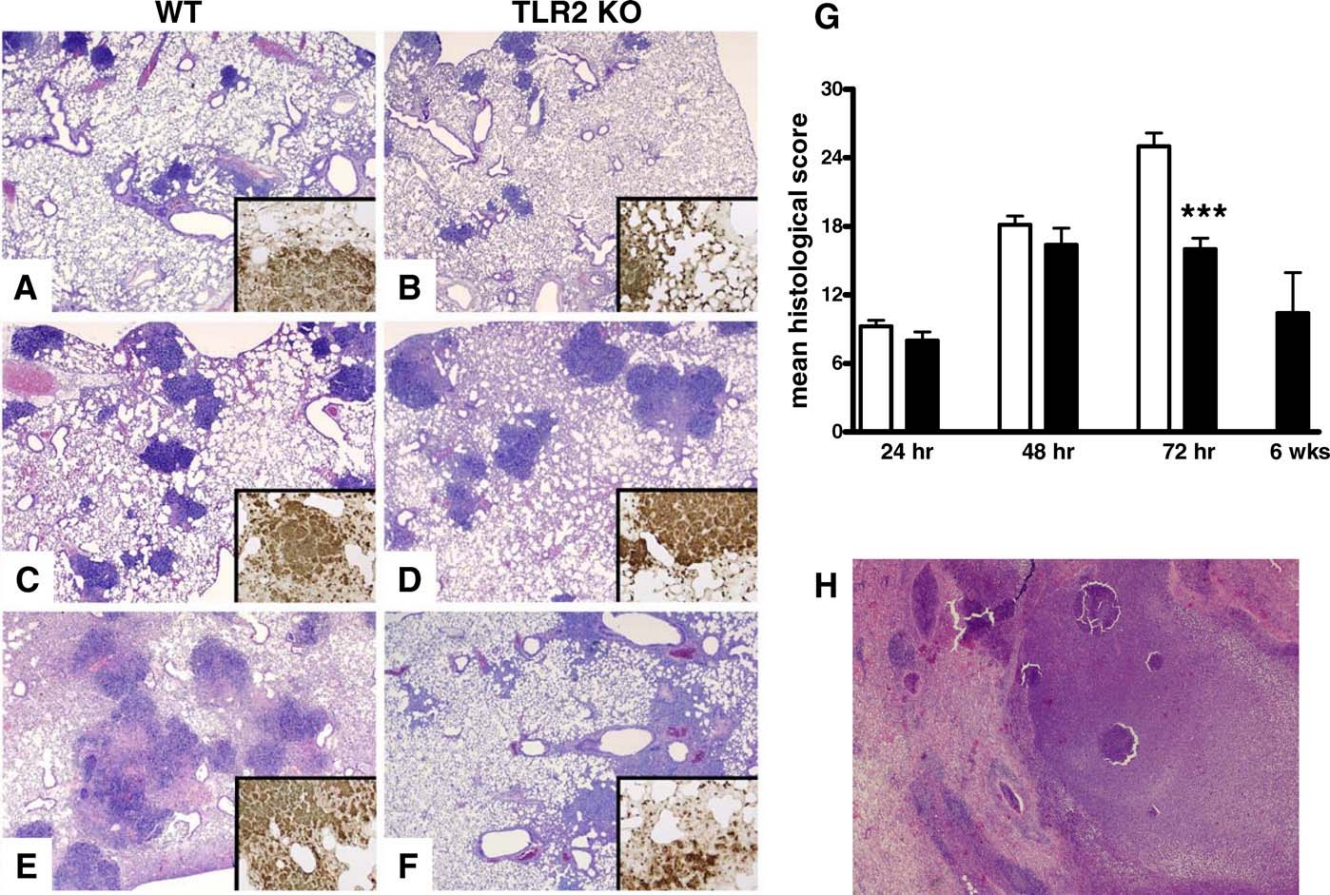
Reduced Lung Inflammation in TLR2 KO Mice 72 Hours after Infection Representative lung histology of WT (A, C, and E) and TLR2 KO mice (B, D, and F) at 24 h (A and B), 48 h (C and D), and 72 h (E and F) after inoculation with 5 × 10^2^ CFU *B. pseudomallei.* TLR2 KO showed significantly less inflammation, pleuritis, peribronchial inflammation, edema, endothelialitis, and necrosis 72 h after infection in the mice (F and G) compared to WT (E–G). Pathology scores (mean ± SEM) (G) were calculated as described in the Methods section; open bars represent WT mice, and solid bars represent the TLR2 KO mice. The insets (A–F) are representative images of immunostaining for granulocytes, showing dense granulocytic infiltrations and confirming reduced inflammation and granulocyte influx in the TLR2 KO mice at 72 h after inoculation with B. pseudomallei. TLR2 KO mice that survived the experiment shown in Figure 3A were examined 6 wk after inoculation; as expected, they showed decreased organ inflammation as compared to the 72 h time point The abscesses that were still present were encapsulated (H). Magnification, 20×. All data are from eight mice per group at each time point, except for (H) (*n* = 5).

To obtain further insight into the involvement of TLR2 and TLR4 in the inflammatory response after infection with B. pseudomallei, we semiquantitatively scored lung histology slides generated from WT, TLR2 KO, and TLR4 KO mice at various time points after infection. Infection of WT mice with B. pseudomallei was characterized by severe pulmonary inflammation, with abscess formation, necrosis, endothelialitis, and thrombus formation. Consistent with the observed reduced bacterial outgrowth, TLR2 KO mice displayed reduced organ inflammation at 72 h postinfection when compared with WT mice (Figure 5). Accordingly, the number of neutrophils present in the lung, as analyzed by Gr1-immunostaining, was lower in the TLR2 KO mice (insets, Figure 5A–5F). As expected, TLR2 KO mice that survived the 6 wk survival study described above showed decreased lung inflammation as compared to the 72 h time point; the abscesses that were still present were encapsulated in these animals (Figure 5H). In contrast with the TLR2 KO mice, the TLR4 KO mice showed similar inflammation compared with WT mice (unpublished data).

### Diminished Late Proinflammatory Cytokine and Increased Early Anti-inflammatory Cytokine Production in TLR2 KO Mice

**Figure 6:**
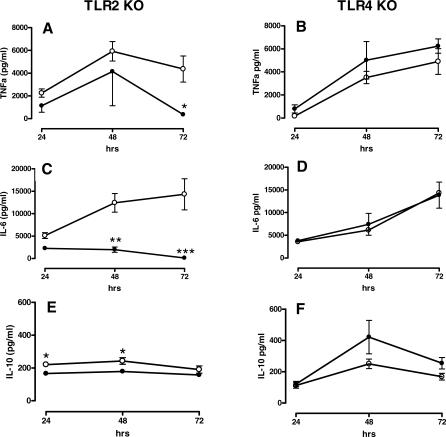
TLR2 KO Mice, but Not TLR4 KO Mice, Show Reduced Pulmonary Cytokine Levels during Experimental Melioidosis WT mice (open symbols) (A–F), TLR2 KO mice (closed symbols) (A, C, and E), and TLR4 KO mice (closed symbols) (B, D, and F) were intranasally infected with 5 × 10^2^ CFU of B. pseudomallei. 24, 48, and 72 h after inoculation mice were sacrificed, lungs were removed, and TNFα (A and B), IL6 (C and D), and IL10 (E and F) were measured in lung homogenates. Data are means ± SEM of eight mice per group per time point. *p < 0.05; **p < 0.001; ***p < 0.0001.

**Figure 7:**
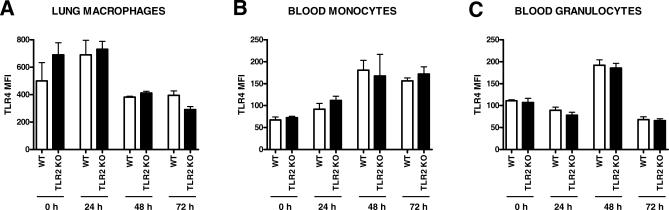
No Difference in TLR4 Expression in TLR2 KO Mice Compared to WT Mice during Infection with B. pseudomallei TLR4 cell surface expression is depicted over time (0, 24, 48, and 72 h after intranasal inoculation with B. pseudomallei), on the lung macrophages (A), blood monocytes (B), and blood granulocytes (C). Data are expressed as mean ± SEM and *n* = 4 mice per group at each time point. MFI, mean fluorescence intensity.

The success of combating infections in the lung strongly depends on the efficacy of the local inflammatory response elicited [29,30]. In order to study the extent and kinetics of the inflammatory response, we sacrificed WT, TLR2 KO, and TLR4 KO mice at multiple time points after infection and measured the concentrations of the proinflammatory cytokines TNFα and IL6 and the anti-inflammatory cytokine IL10 in lung homogenates (Figure 6). TNFα and IL6 levels were lower in TLR2 KO mice than in WT mice, especially at 72 h after infection (*p* < 0.05 and *p* < 0.001, respectively). In addition, lung IL10 concentrations were moderately lower in TLR2 KO mice at 24 and 48 h after infection (*p* < 0.05). In contrast, the pulmonary levels of these mediators were similar in TLR4 KO and WT mice at all time points. In addition, TLR2 deficiency did not influence the expression of TLR4 on lung macrophages or blood monocytes or granulocytes throughout the infection (Figure 7).

### TLR2 KO Mice, but Not TLR4 KO Mice, Demonstrate Decreased Distant Organ Injury

**Figure 8:**
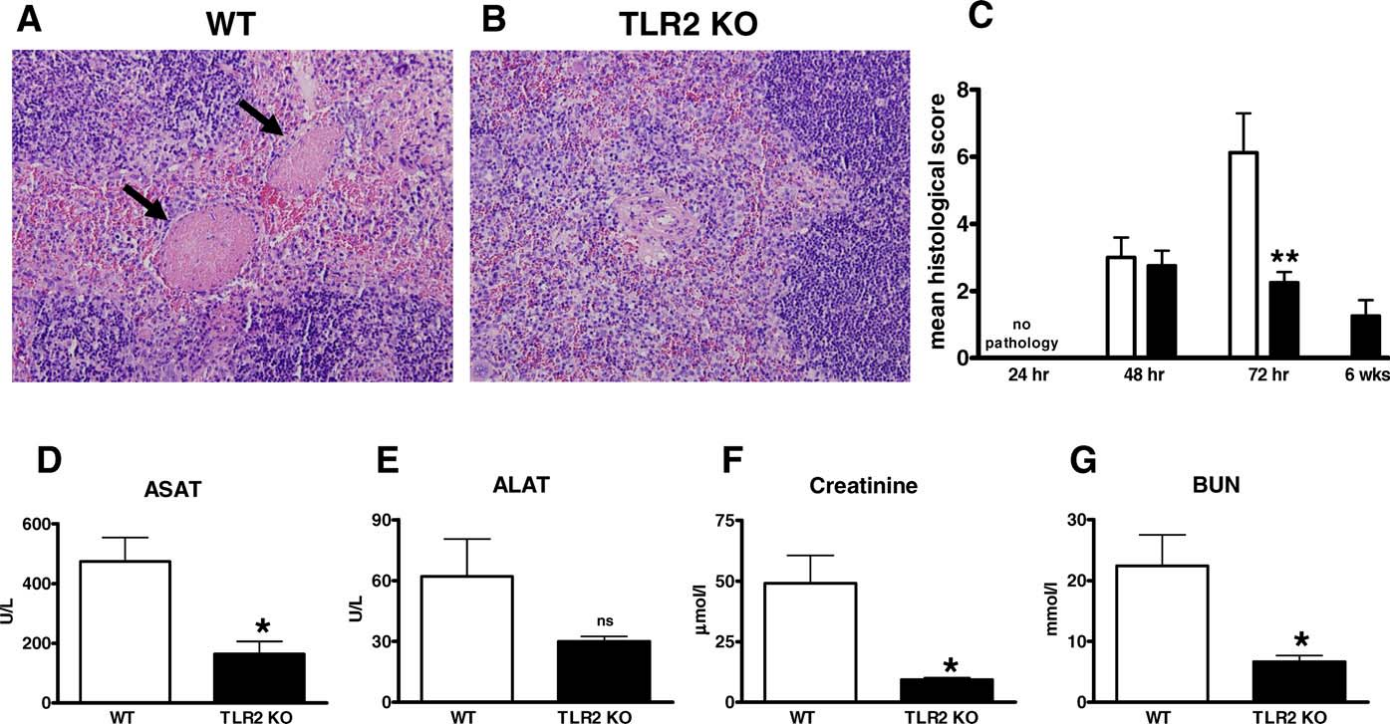
TLR2 KO Mice Demonstrate Reduced Distant Organ Injury Mice were intranasally inoculated with 5 × 10^2^ CFU of B. pseudomallei. After 72 h, representative spleen histology of WT (A) and TLR2 KO (B) mice demonstrated decreased inflammation and less thrombus formation in the TLR2 KO spleens. Magnification, 12.5×. Arrows indicate increased thrombus formation in the WT mice compared to TLR2 KO mice. The reduced inflammation and injury in the spleen at 72 h after infection (C) was confirmed by the semiquantitative pathology score described in the Methods. At this time point TLR2 KO mice also showed less hepatic injury, as reflected by the plasma concentrations of ASAT (D) and ALAT (E), and less renal failure, as reflected by plasma creatinine (F) and blood urea nitrogen (BUN) (G). Data are expressed as mean ± SEM of eight WT mice (open bars) and eight TLR2 KO mice (black bars). *p < 0.05; **p < 0.01 versus WT control.

To further evaluate the role of TLR2 and TLR4 in the systemic inflammatory response after infection with B. pseudomallei, we semiquantitatively scored spleen histology slides generated from infected mice and performed routine clinical chemistry to evaluate hepatic and renal injury. No differences were observed between WT and TLR4 KO mice at any time point (unpublished data). However, TLR2 KO showed diminished inflammation in their spleens when compared to the WT mice (Figure 8A–8C). Furthermore, liver and kidney function were relatively well preserved in TLR2 KO mice, as reflected by lower ASAT, ALAT, blood urea nitrogen (BUN), and creatinine plasma levels in these animals (Figure 8D–8G).

## Discussion

In the present study we aimed to characterize the expression and function of the TLRs in septic melioidosis, linking observational studies in patients with culture-proven disease with functional studies in TLR-deficient mice. We made the following key observations. (1) Patients with septic melioidosis have increased expression of CD14, TLR1, TLR2, and TLR4 on the cell surfaces of circulating monocytes and granulocytes and increased levels of mRNAs encoding CD14, LY96, TLR1, TLR2, TLR3, TLR4, TLR5, TLR8, and TLR10 in peripheral blood cells. (2) Both TLR2 and TLR4 contribute to cellular responsiveness to *Burkholderia* in vitro, as reflected by reduced TNFα release by alveolar macrophages and whole blood from TLR2 KO and TLR4 KO mice and by activation of HEK293 cells stably transfected with either TLR2 or TLR4 upon incubation with B. pseudomallei. (3) The LPS of B. pseudomallei signals through TLR2 and not through TLR4. And (4) TLR4 is not important for protective immunity against experimentally induced melioidosis, whereas TLR2 is associated with the growth and dissemination of the infection, significantly contributing to distant organ injury and lethality.

Our study is, to our knowledge, the first to provide insights into the relative gene expression of all TLR family members in peripheral blood monocytes and granulocytes in patients with severe sepsis. In blood samples obtained from 34 prospectively enrolled patients with sepsis caused by B. pseudomallei, we showed that a whole repertoire of TLRs is up-regulated at the mRNA level in both monocytes and granulocytes. Of note, patients showed enhanced monocyte and granulocyte expression of three of four TLR4 splice variants. TLR4 variant R02 remained undetectable, which is consistent with our earlier study examining alveolar macrophages from healthy humans challenged with LPS via the airways [15]. TLR4 variant R01 is involved in LPS binding and triggering of intracellular signal transduction cascades, while variants R03 and R04 lack residues 24–34, which are essential for LY96 (MD2) binding and LPS signaling [31]. The expression of TLR3 mRNA was very low in both patients and healthy controls, which can be explained by the fact that this intracellular receptor is primarily expressed by dendritic cells and to a lesser extent B and T cells [32–34]. In addition, we could not detect *TLR9* mRNA in monocytes or granulocytes, which is in line with studies showing that *TLR9* mRNA in particular is expressed in plasmacytoid dendritic cells and B cells. The increase in mRNAs encoding CD14, TLR1, TLR2, and TLR4 was accompanied by enhanced surface expression of the respective proteins circulating neutrophils and granulocytes. This is in accordance with two earlier clinical reports, which found increased expression of TLR2 and TLR4 on neutrophils and monocytes from septic patients [35,36].

Humans usually acquire melioidosis by inoculation through skin abrasions or inhalation [3,4]. Pneumonia with bacterial dissemination to distant body sites is a common presentation of melioidosis [5]. We decided to develop a mouse model in which B. pseudomallei was administered via the airways, since this route may be more clinically relevant than the intraperitoneal route used in several previous studies. We reproduced the major clinical characteristics of melioidosis, with rapid spread of bacteria to distant organs, multiple organ failure and abscess formation. Among the TLRs with enhanced expression in patients with melioidosis, we considered TLR2 and TLR4 most interesting with respect to the impact of their loss of function in experimentally induced melioidosis in mice. Indeed, of the other TLRs that showed increased expression, TLR1 has been implicated as a co-receptor together with TLR2 in the recognition of bacterial triacyl lipopeptides, whereas TLR8 senses single-stranded viral RNA [1,2]. In addition, although TLR5, as the receptor that recognizes flagellin, in theory may be involved in host defense against *B. pseudomallei,* a recent report suggests that TLR5 especially or exclusively senses pathogenic flagellated bacteria present in the intestinal lumen, whereas the expression of this receptor was low in the mouse lung [37].

A remarkable and unexpected finding was that, although TLR4 appeared very important for cellular responsiveness to B. pseudomallei in vitro (Figure 2), this receptor does not significantly contribute to host defense against melioidosis in vivo. In line with these in vivo observations, we demonstrated that purified LPS from B. pseudomallei 1026b (the strain also used in the in vivo infection studies) activates HEK293 cells via TLR2, not via TLR4. The evidence that TLR4 has an important role in gram-negative infections is exclusively derived from models using bacteria that express LPS recognized by TLR4 [9]. Indeed, TLR4-deficient mice are more susceptible to gram-negative sepsis due to Salmonella typhimurium and Escherichia coli and to gram-negative pneumonia due to *Haemophilus influenzae, Klebsiella pneumoniae,* and Acinetobacter baumannii [27,38–41]. Of note, B. pseudomallei LPS differs in several aspects from the LPS of other gram-negative organisms: B. pseudomallei LPS exhibits weaker pyrogenic activity in rodents compared with enterobacterial LPS [4,42], and LPS-mediated activation of mouse macrophages in vitro is slower for LPS from B. pseudomallei than for E. coli LPS [43]. In this respect it is of interest that our laboratory previously established that TLR4 is not involved in host defense against pulmonary infection with Legionella pneumophila [44], a gram-negative bacterium of which the LPS, like B. pseudomallei LPS, is a weak inducer of proinflammatory cytokine production by mononuclear cells [45]. Hence, it is conceivable that absent or insufficient LPS sensing by TLR4 makes this receptor redundant during infection in vivo with *Legionella* or *Burkholderia*. In addition, the recent finding that TLR4 does not mediate resistance against acute lung infection due to *Pseudomonas aeruginosa* suggests that for some gram-negative pathogens signaling to other pattern recognition receptors may compensate for the loss of LPS–TLR4 signaling [46].

TLR2 is a promiscuous pattern recognition receptor recognizing multiple ligands expressed by a variety of microorganisms such as *Mycoplasma* (diacyl lipopeptides), mycobacteria (triacyl lipopeptides, lipoarabinomannan), fungi (zymosan, phospholipomannan), parasites (tGPI-mutin), and viruses (hemagglutinin protein from measles virus and unidentified structures of herpes simplex virus) [1,2]. In addition, TLR2 can signal a number of PAMPs expressed by bacteria, in particular lipoteichoic acid (exclusively expressed by gram-positive bacteria), peptidoglycan and triacyl lipopeptides (found in most bacteria), and porins *(Neisseria)* [1,2]. Finally, TLR2 may be activated by LPS from some gram-negative bacteria [47–49]. We here found that TLR2 contributes to the responsiveness of cells to B. pseudomallei using two complementary approaches: TLR2 KO macrophages and blood produced less TNFα upon stimulation with *Burkholderia*, whereas TLR2 transfected HEK293 cells released large amounts of IL8 upon incubation with this bacterium. In addition, we identified B. pseudomallei LPS as a TLR2 ligand. Conceivably, B. pseudomallei expresses more TLR2 ligands, with peptidoglycan and lipopeptides being possible candidates. The interaction between *Burkholderia* and TLR2 clearly impairs the protective immune response against this bacterium, as reflected by the fact that TLR2 KO mice displayed a strongly reduced mortality during experimental melioidosis, which was associated with a diminished bacterial growth and dissemination, and reduced distant organ injury. Previously, TLR2 KO mice were found to be less susceptible to lethal infections with Yersinia enterocolitica or Candida albicans through a mechanism that likely involved a stronger type 1 cytokine response due to diminished production of the prototypic type 2 cytokine IL10 during infection [50,51]. In the case of *Candida* infection, TLR2 induced proliferation and survival of regulatory T cells, which appeared largely responsible for the increased IL10 release induced by TLR2 [51]. Although a type 1 response is protective in melioidosis [52,53], a loss of type 2 cytokine stimulation in TLR2 KO mice cannot explain the protection of these animals during B. pseudomallei infection. First, the differences in IL10 concentrations between TLR2 KO and WT mice were minor and much less profound than the concurrent differences in the proinflammatory cytokine TNFα. Second, the concentrations of the prototypic type 1 cytokine interferon gamma remained very low or undetectable throughout the entire observation period (unpublished data). Finally, antibody-mediated depletion of regulatory T cells did not influence the outcome of melioidosis in our model (unpublished data). More studies are warranted to dissect the exact mechanism by which TLR2 facilitates the growth and spread of B. pseudomallei in vivo.

Melioidosis is a debilitating disease with an unacceptably high mortality rate [3,4]. We here demonstrate that the expression of multiple TLRs is up-regulated in peripheral blood leukocytes in septic patients with melioidosis. Most remarkable, although our results show that both TLR2 and TLR4 contribute to cellular responsiveness to B. pseudomallei in vitro, TLR2 detects the LPS of B. pseudomallei and only TLR2 impacts on the immune response of the intact host in vivo. Of note, TLR4 does not contribute to protective immunity in this severe gram-negative infection. Taken together, these findings suggest that septic melioidosis can be seen as the clinical manifestation of a TLR-mediated dysregulation of the immune response to invasive B. pseudomallei. In addition, this report further undermines the paradigm regarding TLR4 as “the gram-negative receptor” and TLR2 as “the gram-positive receptor.” Inhibition of TLR2 may be a useful adjunctive therapy for melioidosis.

## Supporting Information

Alternative Language Abstract S1 Translation of the abstract into Thai by Direk Limmathurotsakul (74 KB PDF)

## Abbreviations

ALAT: alanine aminotransferase
ASAT: aspartate aminotransferase
CFU: colony-forming unit(s)
IL: interleukin
KO: knockout
LPS: lipopolysaccharide
MFI: mean fluorescence intensity
MLPA: multiplex ligation-dependent probe amplification
PAMP: pathogen-associated molecular pattern
SEM: standard error of the mean
TLR: Toll-like receptor
TNFα: tumor necrosis factor alpha
WT: wild type
