# Supplementary material for: Toll-Like Receptor 2 Impairs Host Defense in Gram-Negative Sepsis Caused by Burkholderia pseudomallei (Melioidosis)
Source: PLoS Med. 2007 Jul 31;4(7):e248. doi: 10.1371/journal.pmed.0040248 (PMC1950213; doi:10.1371/journal.pmed.0040248)
Supplement: Alternative Language Abstract S1 — (74 KB PDF) [file pmed.0040248.sd001.pdf]

ภูมิคุ้มกันบกพร่องจาก TOLL-LIKE RECEPTOR 2 ในผู้ป่วยเมลิออยโดสิส

W. Joost Wiersinga[1,2], Cathrien W. Wieland[1,2], Mark C. Dessing[1,2], Narisara Chantratita[4], Allen C. Cheng[5], Direk Limmathurotsakul[4], Wirongrong Chierakul[4], Masja Leendertse[1,2], Sandrine Florquin[3], Alex F. de Vos[1,2], Nicholas White[4,6], Arjen M. Dondorp[4], Nicholas P. Day[4,6], Sharon J. Peacock[4,6], Tom van der Poll[1,2]*

[1]Center for Infection and Immunity Amsterdam (CINIMA), [2]Center for Experimental and Molecular Medicine, and [3]Department of Pathology, Academic Medical Center, Amsterdam, the Netherlands, [4]Wellcome Trust, Mahidol University, Bangkok, Thailand, [5]Menzies School of Health Research, Darwin, Australia and [6]Center for Clinical Vaccinology and Tropical Medicine, University of Oxford, Oxford, England.

บทคัดย่อ

บทนำ

Toll-like receptors (TLRs) มีบทบาทสำคัญในระบบภูมิคุ้มกันตามธรรมชาติที่มีมาแต่กำเนิด และเกี่ยวข้องกับการจดจำลักษณะของเชื้อโรคและกระตุ้นให้เกิดการต่อต้านเชื้อแบคทีเรีย โดยพบว่า TLR2 สำคัญที่สุดในการติดเชื้อแกรมบวก และ TLR4 สำคัญที่สุดในการติดเชื้อแกรมลบ เมลิออยโดสิส เป็นโรคติดเชื้อที่มีความรุนแรงเกิดจากเชื้อแกรมลบ *Burkholderia pseudomallei* ที่พบในเอเชียตะวันออกเฉียงใต้ การศึกษานี้เพื่อดูการทำงานและการแสดงออกของ TLRs ในผู้ป่วยเมลิออยโดสิสที่ติดเชื้อรุนแรง

วิธีการศึกษาและผลการศึกษา

จากการศึกษาผู้ป่วยเมลิออยโดสิสจำนวน 34 ราย พบว่ามีการแสดงออกของ CD14, TLR1, TLR2 และ TLR4 บนผิวเซลล์ของโมโนซัยท์ และแกรนูโลซัยท์เพิ่มขึ้น และพบว่ามีระดับ mRNA ของ CD14, TLR1, TLR2, TLR4, MD-2, TLR5 และ TLR10 ในโมโนซัยท์และแกรนูโลซัยท์ที่แยกมาจากผู้ป่วยมากขึ้นเมื่อเทียบกับกลุ่มอาสาสมัครที่แข็งแรง

ผลการทดลองในหลอดทดลองพบว่า ในหนูทดลองที่ขาด TLR2 และ TLR4 เลือดและแมคโครฟาจในปอด มีการตอบสนองต่อเชื้อ *B. pseudomallei* น้อยลง ในขณะที่เซลล์เพาะเลี้ยงไตอ่อน (HEK 293)ซึ่งปกติไม่ตอบสนองต่อเชื้อ *B. pseudomallei* แต่เมื่อทำให้เซลล์ติดเชื้อไวรัสที่เป็นพาหะของ TLR2 หรือ TLR4 กลับทำให้มีการตอบสนองต่อเชื้อ *B. pseudomallei* ได้ นอกจากนั้นไลโปโพลีแซคคาไรด์จากผนังเซลล์ของเชื้อ *B. pseudomallei* สามารถกระตุ้นกลไกของภูมิคุ้มกันผ่าน TLR2 แต่ไม่ผ่านทาง TLR4

การศึกษาในหนูที่มีพันธุกรรมชนิดที่ไม่มีการสร้าง TLR4 พบว่าไม่มีความแตกต่างจากหนูปกติทั้งในด้านจำนวนเชื้อที่พบในหนูและอัตราการเสียชีวิตในช่วงการศึกษา ในขณะที่หนูที่มีพันธุกรรมชนิดที่ไม่มีการสร้าง TLR2 มีการตอบสนองของภูมิคุ้มกันดีมากขึ้น โดยเห็นได้จากอัตราการรอดชีวิตที่สูงกว่า รวมถึงพบปริมาณเชื้อในหนู การอักเสบของปอดและเนื้อเยื่ออื่นๆน้อยกว่าที่พบในหนูปกติที่มีการติดเชื้อ

สรุป

ผู้ป่วยโรคเมลิออยโดสิสมีการกระตุ้นโมโนซัยท์และแกรนูโลซัยท์ให้มีการเพิ่ม TLRs หลายชนิด และถึงแม้ว่าทั้ง TLR2 และ TLR4 จะมีส่วนในการตอบสนองต่อเชื้อ *B. pseudomallei* จากการศึกษาในหลอดทดลอง แต่เฉพาะ TLR2 เท่านั้นที่สามารถตรวจจับไลโปโพลีแซคคาไรด์ ของเชื้อ *B. pseudomallei* และมีผลกระทบกับระบบภูมิคุ้มกันในร่างกาย ดังนั้นการยับยั้ง TLR2 อาจเป็นแนวทางใหม่ในการช่วยรักษาโรคติดเชื้อเมลิออยโดสิส
